# A prospective neurosurgical registry evaluating the clinical care of traumatic brain injury patients presenting to Mulago National Referral Hospital in Uganda

**DOI:** 10.1371/journal.pone.0182285

**Published:** 2017-10-31

**Authors:** Benjamin J. Kuo, Silvia D. Vaca, Joao Ricardo Nickenig Vissoci, Catherine A. Staton, Linda Xu, Michael Muhumuza, Hussein Ssenyonjo, John Mukasa, Joel Kiryabwire, Lydia Nanjula, Christine Muhumuza, Henry E. Rice, Gerald A. Grant, Michael M. Haglund

**Affiliations:** 1 Duke University Division of Global Neurosurgery and Neurology, Durham, North Carolina, United States of America; 2 Duke University Global Health Institute, Durham, North Carolina, United States of America; 3 Duke-National University Singapore Medical School, Singapore, Singapore; 4 Stanford University School of Medicine, Palo Alto, California, United States of America; 5 Stanford Center for Innovation in Global Health, Palo Alto, California, United States of America; 6 Department of Emergency Medicine, Duke University Medical Center, Durham, North Carolina, United States of America; 7 Department of Neurosurgery, Stanford University Medical Center, Palo Alto, California, United States of America; 8 Department of Neurosurgery, Mulago Hospital, Kampala, Uganda; 9 Makerere University School of Public Health, Kampala Uganda; 10 Department of Surgery, Duke University Medical Center, Durham, North Carolina, United States of America; 11 Department of Neurosurgery, Duke University Medical Center, Durham, North Carolina, United States of America; University Health Network and University of Toronto, CANADA

## Abstract

**Background:**

Traumatic Brain Injury (TBI) is disproportionally concentrated in low- and middle-income countries (LMICs), with the odds of dying from TBI in Uganda more than 4 times higher than in high income countries (HICs). The objectives of this study are to describe the processes of care and determine risk factors predictive of poor outcomes for TBI patients presenting to Mulago National Referral Hospital (MNRH), Kampala, Uganda.

**Methods:**

We used a prospective neurosurgical registry based on Research Electronic Data Capture (REDCap) to systematically collect variables spanning 8 categories. Univariate and multivariate analysis were conducted to determine significant predictors of mortality.

**Results:**

563 TBI patients were enrolled from 1 June– 30 November 2016. 102 patients (18%) received surgery, 29 patients (5.1%) intended for surgery failed to receive it, and 251 patients (45%) received non-operative management. Overall mortality was 9.6%, which ranged from 4.7% for mild and moderate TBI to 55% for severe TBI patients with GCS 3–5. Within each TBI severity category, mortality differed by management pathway. Variables predictive of mortality were TBI severity, more than one intracranial bleed, failure to receive surgery, high dependency unit admission, ventilator support outside of surgery, and hospital arrival delayed by more than 4 hours.

**Conclusions:**

The overall mortality rate of 9.6% in Uganda for TBI is high, and likely underestimates the true TBI mortality. Furthermore, the wide-ranging mortality (3–82%), high ICU fatality, and negative impact of care delays suggest shortcomings with the current triaging practices. Lack of surgical intervention when needed was highly predictive of mortality in TBI patients. Further research into the determinants of surgical interventions, quality of step-up care, and prolonged care delays are needed to better understand the complex interplay of variables that affect patient outcome. These insights guide the development of future interventions and resource allocation to improve patient outcomes.

## Introduction

Annual global death from injury is estimated to be 5 million, of which 90% occur in low- and middle-income countries (LMICs).[1,2] Rising injury mortality can be attributed in part to the 50% increase in road traffic injuries (RTI) over the past two decades, now accounting for a quarter of deaths from injury.[1] Furthermore, this estimation is likely to increase by the year 2030 when RTI is projected to become the fifth leading cause of death.[3] Referred to as the “silent epidemic”[4], traumatic brain injury (TBI) is the most critical sequelae of RTIs with the greatest contribution to mortality and disability adjusted life years (DALYs).[5]

The global incidence of TBI from RTIs is disproportionately concentrated in LMICs with rates of 150–170 per 100,000 TBI in Sub Saharan Africa (SSA) compared to 106 per 100,000 globally.[6] Furthermore, this burden is compounded by greater risk factors and limited healthcare capacity in LMICs.[5] In a study comparing mortality from injury between Kampala Uganda and the United States, 25% of all deaths in Kampala were due to injuries compared to 7% in the United States, and the odds of dying from injury were 4.2 times higher in Uganda.[7]

The disparities in the injury incidence and outcome between LMICs and resource-rich settings have led to increased health outcomes research for TBIs and their associated risk factors in LMICs.[8,9] While there have been increasing TBI studies in LMICs over the last decade, there is still a need for more robust prospective registries.[10] In Uganda, a trauma registry implemented in 2004 at the Mulago National Referral Hospital (MNRH) showed that RTI is the major contributor (60%) of overall mortality in the casualty department.[10] Within the critical care department, TBI was the most common admission diagnosis and contributed to an overall mortality rate of 44%.[11] In another analysis evaluating severe TBI patients, the mortality rate was 25.8%.[12] While the prior registry provides information on injury incidence and burden, it’s limited in scope and doesn’t follow patients longitudinally throughout their hospital stay nor does it focus specifically on TBIs. And although these retrospective analyses are helpful for benchmarking TBI outcomes, they make it hard to identify specific quality improvement initiatives. The relationship among epidemiology, patient risk factors, clinical care, and TBI outcomes are still relatively unknown at MNRH. The objectives of this study are to describe the processes of care and determine risk factors predictive of poor outcomes for TBI patients presenting to a single tertiary hospital in Uganda.

## Materials and methods

### Institutional review board

Ethical approval was provided by the Duke University and Stanford University Institutional Review Boards (IRB) in the U.S. (protocols 69190 and 34346, respectively) as well as the Mulago National Referral Hospital Research and Ethics Committee in Uganda (protocol 1020).

### Study design

**Fig 1:**
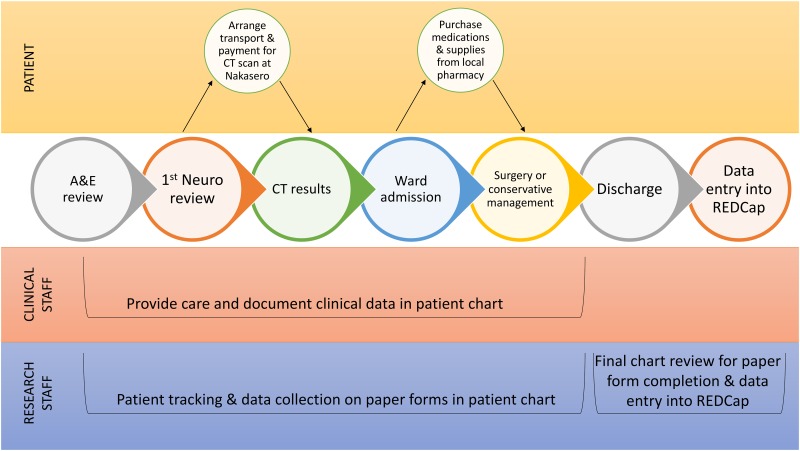
Mulago National Referral Hospital (MNRH) traumatic brain injury (TBI) prospective rapid electronic data capture (REDCap) registry workflow.

A prospective registry of TBI patients was created using Research Electronic Data Capture (REDCap). TBI was defined by clinical and/or radiological evidence of head injury alone or in association with other injuries. All patients presenting to the emergency department at MNRH referred to the neurosurgery team with a documented TBI diagnosis were included in this study. TBI severity was categorized based on admission GCS into the following: mild (GCS 13–15), moderate (GCS 9–12), severe (GCS 6–8, GCS 3–5). In-country research assistants (RAs) reviewed patient charts and tracked patients throughout their hospital stay by observing daily rounds. Printed forms of the registry variables were added to the patient charts and filled by the RAs throughout the patient’s hospital stay. Data entry into the REDCap registry was completed at the end of the patient’s hospital stay (Fig 1). Data quality was assessed among the research team every 4 weeks, and discrepancies were clarified with the RAs.

### Variables

A total of 60 variables were tracked in the registry and divided into 8 categories: demographics, patient baseline and risk factors of in-hospital mortality, clinical assessment, diagnostics, management, care continuum, complications, and discharge status. Relevant risk factor variables were determined from prior studies involving the National Trauma Databank.[13] Clinical assessment and diagnostics were recorded as described in the patient chart.

### Statistical analysis

Statistical analyses were performed using Stata version 14.0 (Stata Corp, College Station, TX). Univariate analysis of association between variables with mortality was performed using cross-tabulation, chi square, and Fisher’s exact test when appropriate. Risk factors significant at the p < 0.05 level on univariate analysis were entered into the multivariate model for logistic regression using multivariate analysis. The final multivariate model was constructed through stepwise backward elimination until all the remaining variables were significant at the p < 0.05 level. This approach was taken because our sample was restricted to be able to produce a model with the many possible risk factors studied. Therefore, we chose to perform stepwise elimination to produce the most parsimonious model that would also be informative considering the limitations in implementing a new registry in a LMIC. Nevertheless, clinically relevant confounders were controlled for during the modelling process and they include age, presence of polytrauma, and CT diagnosis. Total cases, mortality counts, univariate p-values, unadjusted odds ratios (OR), adjusted OR, and 95% confidence intervals (CI) were reported.

## Results

### Outcomes

**Fig 2:**
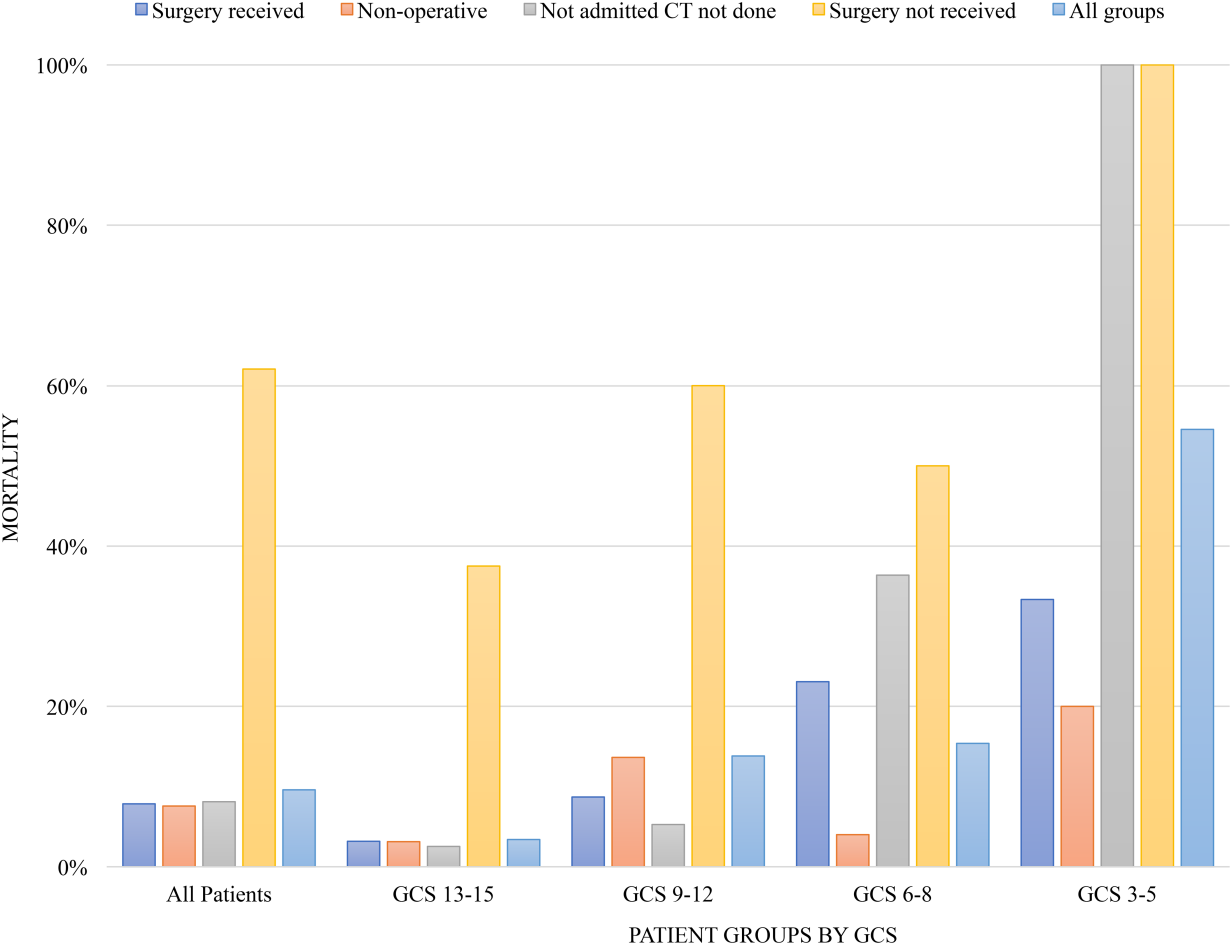
Mortality of traumatic brain injury (TBI) patients presenting to Mulago National Referral Hospital (MNRH) by traumatic brain injury (TBI) severity and management pathway.

The sample size of the study was 563 with an overall mortality rate of 9.6% (54 cases) (Fig 2). Eight died after surgery, 18 died while awaiting surgical intervention (per the patient chart), 19 died during non-operative management, and 9 died prior to CT done and admission. Mortality rate for mild TBI was 3.4% and increased to 13.8%, 15%, and 55% for moderate and severe TBI (admission GCS 6–8, GCS 3–5), respectively. Highest rates of death were seen in patients who failed to receive surgery and those without CT results.

### Patient demographics

During the 6-month period from 1 June 2016 to 30 November 2016, 563 patients met the inclusion criteria to be enrolled in the TBI registry at MNRH. The average age was 29 (IQR 20–36), and mortality increased by age group from 5% in the 0–14 age group to 17% in the above 45 age group (Table 1). More than 70% of patients were in the 15–44 age group and 86% were male. The most common cause of TBI was road traffic injury (62%), of which most were pedestrians and motorcyclists. Half of the patients had less than a secondary education, and 43% were referred from a primary care center.

**Table 1:** TBI patient demographic variable association with mortality.

Demographics	Total Cases N = 563 (%)	Alive N = 509 (%)	Died N = 54 (%)	Univariate p-Value	Unadjusted OR (95% CI)
Age (years)					
0–14	78 (13.8)	74 (94.9)	4 (5.1)	Ref	
15–29	243 (43.4)	225 (92.6)	18 (7.4)	0.510	1.45 (0.50–4.51)
30–44	160 (28.3)	143 (89.4)	17 (10.6)	0.170	2.20 (0.71–6.77)
≥ 45	81 (14.3)	67 (82.7)	14 (17.3)	**0.009**	3.87 (1.21–12.32)
Male gender	488 (86.4)	438 (89.8)	50 (10.3)	0.167	2.09 (0.73–5.97)
Type of injury					
Road traffic injury	350 (62.0)	323 (92.3)	27 (7.7)	Ref	
Assault	136 (24.4)	124 (91.2)	12 (8.8)	0.686	1.16 (0.57–2.36)
Fall	64 (11.3)	52 (81.2)	12 (18.8)	**0.007**	2.76 (1.32–5.79)
Type of road traffic injury					
Pedestrian	192 (55.0)	181 (94.3)	11 (5.7)	Ref	
Motorcycle	96 (27.4)	85 (88.5)	11 (11.5)	0.090	2.13 (0.89–5.11)
Car	16 (4.6)	14 (87.5)	2 (12.5)	0.296	2.35 (0.47–11.66)
Education					
None	40 (20.0)	38 (95.0)	2 (5.0)	0.315	2.32 (0.24–9.29)
Primary	60 (30.0)	57 (95.0)	3 (5.0)	0.622	1.51 (0.29–7.65)
Secondary	88 (44.0)	85 (96.6)	3 (3.4)	Ref	
University and above	13 (6.4)	12 (92.3)	1 (7.7)	0.466	2.39 (0.23–24.57)
Occupation					
Unemployed	74 (29.5)	70 (94.6)	4 (5.4)	Ref	
Self employed	180 (71.7)	168 (93.3)	12 (6.7)	0.707	3.65 (0.34–38.56)
Formal employment	58 (23.1)	53 (91.4)	5 (8.6)	0.471	1.75 (0.33–5.79)
Primary care referral	242 (42.8)	222 (91.7)	20 (8.3)	0.630	0.87 (0.43–1.39)

Total case column percentages determined using total cases (N = 563) as denominator. Alive and died column percentages determined using total case number for respective variable. The reference group for type of injury is road traffic injury and within the road traffic injury group, pedestrian is the reference group.

### Diagnostics

Documented admission vitals (HR, RR, BP, Temperature) and lab testing in the patient chart were seen in 95 out of 563 patients (17%), most of which were patients admitted for surgical intervention (Table 2). CT results were available for 440 out of 563 patients (78%), of which 9.3% were normal results. Intracranial hemorrhages diagnosed in descending order were: epidural (18%), acute subdural (15%), parenchymal (5.5%), intraventricular (1.8%), subarachnoid (3.6%). An additional 5.7% had more than one intracranial hemorrhage. Non-intracranial hemorrhage pathologies represented nearly 40% of CT findings: fracture (9.3%), edema (4.5%), contusion (12%), and more than one of the above without intracranial bleed (13%). Nearly all (98.6%) had documented admission GCS, cranial nerve and neuro examinations.

**Table 2:** Diagnostic variable association with mortality.

Diagnostic	Total Cases N (%)	Alive N (%)	Died N (%)	Univariate p-Value	Unadjusted OR (95% CI)
Total patient	563 (100.0)	509 (90.4)	54 (9.6)		
Admission vitals recorded	95 (16.8)	86 (90.5)	9 (9.5)	0.685	1.18 (0.59–2.90)
Laboratory CBC done	95 (16.8)	87 (91.6)	8 (8.4)	0.449	1.34 (0.44–2.27)
CT done	440 (77.9)	397 (90.2)	43 (9.8)		
Normal	41 (9.3)	40 (97.6)	1 (2.4)	Ref	
Acute subdural hematoma	66 (15.0)	56 (84.8)	10 (15.2)	**0.044**	8.00 (1.30–58.06)
Epidural hematoma	79 (18.0)	71 (89.9)	8 (10.1)	0.199	4.04 (0.54–37.35)
Parenchymal hematoma	24 (5.5)	23 (95.8)	1 (4.2)	0.665	1.86 (0.10–29.14)
Intraventricular hematoma	8 (1.8)	8 (100.0)	0 (0)	NA	NA
Subarachnoid hematoma	16 (3.6)	13 (81.3)	3 (18.8)	0.069	9.23 (0.88–96.60)
More than one intracranial hemorrhage	25 (5.7)	18 (72.0)	7 (28.0)	**0.007**	18.80 (1.78–135.95)
Fracture only	41 (9.3)	41 (100.0)	0 (0)	NA	NA
Edema only	20 (4.5)	20 (100.0)	0 (0)	NA	NA
Contusion only	53 (12.0)	50 (94.3)	3 (5.8)	0.702	1.61 (0.25–24.40)
More than one of the following without intracranial bleed: fracture, edema, contusion	57 (13.0)	47 (82.5)	10 (17.5)	**0.033**	8.51 (1.04–69.39)
Midline shift	48 (10.9)	40 (83.3)	8 (16.7)	0.158	1.91 (0.72–4.12)
Basal cistern compressed or absent	44 (10.0)	39 (88.6)	5 (11.4)	0.423	1.55 (0.46–3.77)
Hematoma size big or massive	28 (6.36)	22 (78.6)	6 (21.4)	0.254	0.86 (0.35–2.25)

Total case column percentages determined using total cases (N = 563) as denominator. Alive and died column percentages determined using total case number for respective variable.

CBC, complete blood count; CT, computed tomography.

### Clinical assessment

Mild TBI (GCS 13–15) were seen in 324 patients (57.5%), moderate TBI (GCS 9–12) in 152 patients (27.0%), and severe TBI (GCS 3–8) in 87 patients (15.5%), of which 65 (11.5%) had a GCS 6–8 and 22 (4%) had a GCS 3–5 (Table 3). More than two-thirds of patients had either no documented change in GCS or an improvement of 1 or more between initial and second assessment. Abnormal pupillary findings were seen in 40 (7%).

**Table 3:** Clinical variable association with mortality.

Clinical Variables	Total Cases N (%)	Alive N (%)	Died N (%)	Univariate p-Value	Unadjusted OR (95% CI)
Total patient	563 (100.0)	509 (90.4)	54 (9.6)		
TBI Severity (admission GCS)					
GCS 13–15	324 (57.5)	313 (96.6)	11 (3.4)	Ref	
GCS 9–12	152 (27.0)	131 (86.2)	21 (13.8)	**<0.001**	4.56 (2.14–9.73)
GCS 6–8	65 (11.5)	55 (84.6)	10 (15.4)	**<0.001**	5.17 (2.10–12.76)
GCS 3–5	22 (3.9)	10 (45.5)	12 (54.6)	**<0.001**	34.15 (12.16–80.87)
GCS Change					
No change	195 (34.6)	183 (93.8)	12 (6.2)	Ref	
Improves by 1 or more	196 (34.8)	173 (88.3)	23 (11.7)	0.350	1.73 (0.62–4.03)
Drops by 1	26 (4.6)	23 (88.5)	3 (11.5)	0.680	1.39 (0.52–7.58)
Drops by 2 or more	40 (7.1)	30 (75.0)	10 (25.0)	**0.017**	4.69 (1.33–16.6)
Abnormal Pupillary Exam^a^	40 (7.1)	28 (70.0)	12 (40.0)	**<0.001**	8.51 (3.66–18.07)
Cranial nerve deficit	35 (6.2)	22 (62.9)	13 (37.1)	**<0.001**	7.53 (3.52–16.11)
Limb weakness	61 (10.8)	43 (70.5)	18 (29.5)	**<0.001**	5.70 (2.97–10.93)
Polytrauma	176 (31.3)	157 (89.2)	19 (10.8)	0.438	1.26 (0.68–2.19)
Incontinence	69 (12.3)	47 (68.1)	22 (31.9)	**<0.001**	7.38 (3.93–13.85)

Total case column percentages determined using total cases (N = 563) as denominator. Alive and died column percentages determined using total case number for respective variable.

TBI, traumatic brain injury; GCS, Glasgow coma scale.

^a^Abnormal pupillary exam includes presence of any of the following: anisocoria, pupil dilation, and light reflex deficits

### Patient baseline and risk factors

Risk factors in decreasing order of prevalence were (Table 4): comorbidity (16.5%), open wound (15%), advanced airway support (12%), seizure (11%), oxygen requirement (7%), transfusion (4%), and fever (1%).

**Table 4:** Patient baseline and risk factor association with mortality.

	Total Cases N (%)	Alive N (%)	Died N (%)	Univariate p-Value	Unadjusted OR (95% CI)
Total patient	563 (100.0)	509 (90.4)	54 (9.6)		
Comorbidity	93 (16.5)	82 (88.2)	11 (11.8)	0.464	0.73 (0.31–1.70)
Angina	9 (1.6)	7 (77.8)	2 (22.2)	0.208	2.79 (0.59–14.4)
Oxygen support	39 (6.9)	22 (56.4)	17 (43.6)	**<0.001**	10.13 (4.95–20.72)
Fever	6 (1.1)	5 (83.3)	1 (16.7)	0.592	1.81 (0.22–16.35)
Seizure	63 (11.2)	46 (73.0)	17 (27.0)	0.051	2.33 (1.00–4.63)
Transfusion	21 (3.7)	20 (95.2)	1 (4.8)	0.453	0.47 (0.06–3.47)
Open wound	85 (15.0)	75 (88.2)	10 (11.8)	0.274	1.48 (0.65–2.83)
Advanced airway	66 (11.7)	46 (69.7)	20 (30.3)	**<0.001**	5.92 (3.15–11.11)

Total case column percentages determined using total cases (N = 563) as denominator. Alive and died column percentages determined using total case number for respective variable.

### Care continuum

Nearly a quarter (23%) waited a day before seeking care and 168 (30%) took more than 4 hours to reach MNRH (Table 5). Nearly half (47%) experienced an injury to arrival timeframe longer than 4 hours. The median arrival to review by the neurosurgery team was 2 hours, review to CT result was 17 hours, CT result to surgery was 71 hours, and surgery to discharge was 78 hours.

**Table 5:** Care continuum variable association with mortality.

	Total Cases N (%)	Alive N (%)	Died N (%)	Univariate p-Value	Unadjusted OR (95% CI)
Total patient	563 (100.0)	509 (90.4)	54 (9.6)		
Seeking care: waited at least a day	128 (22.7)	110 (85.9)	18 (14.1)	**0.033**	1.91 (1.10–3.40)
Reaching care: > 4 hours	168 (29.8)	147 (87.5)	21 (12.5)	**0.046**	1.82 (1.10–3.32)
Injury to Arrival					
≤ 4 hours	298 (52.9)	274 (91.9)	24 (8.1)	Ref	
> 4 and ≤ 24 hours	128 (22.7)	113 (88.3)	15 (11.7)	0.232	1.67 (0.77–3.00)
> 1 and ≤ 2 days	37 (6.6)	35 (94.6)	2 (5.4)	0.573	1.13 (0.14–2.88)
> 2 days	74 (13.1)	65 (87.8)	9 (12.2)	0.269	2.11 (0.70–3.56)
Arrival to Neuro review					
≤ 1 hour	239 (42.5)	217 (90.8)	22 (9.2)	Ref	
> 1 and ≤ 4 hours	132 (23.4)	114 (86.4)	18 (13.6)	0.190	1.50 (0.80–3.22)
> 4 hours	189 (33.6)	176 (93.1)	13 (6.9)	0.385	0.76 (0.36–1.49)
Neuro review to CT results					
≤ 4 hour	81 (26.7)	68 (83.9)	13 (16.1)	Ref	
> 4 and ≤ 24 hours	221 (72.9)	207 (93.7)	14 (6.3)	**0.011**	0.35 (0.16–0.79)
> 24 hours	89 (29.4)	80 (89.9)	9 (10.1)	0.253	0.53 (0.24–1.46)
CT results to surgery					
≤ 4 hour	13 (12.7)	9 (69.2)	4 (30.8)	**0.025**	7.20 (1.27–40.68)
> 4 and ≤ 24 hours	15 (14.7)	15 (100.0)	0	NA	NA
> 24 hours	60 (58.8)	56 (93.3)	4 (6.7)	Ref	

Total case column percentages determined using total cases (N = 563) as denominator. Alive and died column percentages determined using total case number for respective variable.

CT, computed tomography

### Management

Surgery was performed for 102 patients (18%) presenting to MNRH with TBI (Table 6) and intended but not provided for 29 patients (5.1%) due to limited operating theatre capacity and delays along the neurosurgical care continuum. Non-operative management was provided to 251 patients (45%). Nearly a third (181 patients, 32.1%) were not admitted from the emergency department, of which 111 (19.7%) did not receive CT testing.

**Table 6:** Management variable association with mortality.

	Total Cases N (%)	Alive N (%)	Died N (%)	Univariate p-Value	Unadjusted OR (95% CI)
Total patient	563 (100.0)	509 (90.4)	54 (9.6)		
Management Pathway					
Surgery received	102 (18.1)	94 (92.2)	8 (7.8)	Ref	
Non-operative	251 (44.6)	232 (92.4)	19 (7.6)	0.887	1.06 (0.41–2.27)
Not admitted CT done	70 (12.4)	70 (100.0)	0	NA	NA
Not admitted CT not done	111 (19.7)	102 (91.9)	9 (8.1)	0.943	1.04 (0.38–2.80)
Surgery not received	29 (5.1)	11 (37.9)	18 (62.1)	**<0.001**	19.23 (6.79–54.45)
HDU received	35 (6.2)	25 (71.4)	10 (28.6)	**<0.001**	6.66 (2.86–15.51)
ICU needed	74 (13.1)	39 (52.7)	35 (47.3)	**<0.001**	24.60 (12.65–47.84)
ICU received	34 (6.0)	16 (47.1)	18 (52.9)	**<0.001**	15.41 (7.25–33.73)
BP and pulse monitoring in ICU	32 (5.7)	16 (50.0)	16 (50.0)	**<0.001**	12.97 (6.02–27.95)
Ventilator support					
Not needed	489 (86.9)	470 (96.1)	19 (3.9)	Ref	
Received	27 (4.8)	11 (40.7)	16 (59.3)	**<0.001**	36.06 (14.75–88.18)
Needed but not received	47 (8.3)	28 (59.7)	19 (41.3)	**<0.001**	17.52 (8.28–36.74)
Ventilator disruption	8 (1.4)	5 (62.5)	3 (37.5)	**0.015**	6.12 (1.42–26.39)
Physiotherapy					
Not needed	504 (89.5)	480 (95.2)	24 (4.8)	Ref	
Received	21 (3.7)	17 (80.9)	4 (19.1)	**0.016**	4.21 (1.25–12.88)
Not adequate or none	38 (6.7)	34 (89.5)	4 (10.5)	0.191	2.11 (0.66–6.13)
Any morbidity	98 (17.4)	83 (84.7)	15 (26.3)	**<0.001**	4.27 (2.17–8.37)

Total case column percentages determined using total cases (N = 563) as denominator. Alive and died column percentages determined using total case number for respective variable.

CT, computed tomography; HDU, high dependency unit; ICU, intensive care unit; BP, blood pressure

Of all 563 TBI patients, 74 patients (13.1%) required intensive care unit (ICU) support, but only 34 patients (6%) received it. ICU availability was predominantly offered to severe TBI patients (57.1%) compared to moderate TBI (32%) and mild TBI (28.6%) cases. Nearly all patients entering ICU (32 out of 34) received continuous BP and pulse monitoring; 27 out of 34 received mechanical ventilation, and 8 of 27 (29.6%) had documented ventilator disruption. 35 patients (6.2%) entered the HDU and only 21 of 59 patients received adequate physiotherapy–completed as requested by the neurosurgical team in the patient chart.

### Predictors of mortality

The two demographic variables associated with mortality on univariate analysis, age ≥ 45 and TBI due to fall, were not retained in the multivariate model (Table 1).

**Fig 3:**
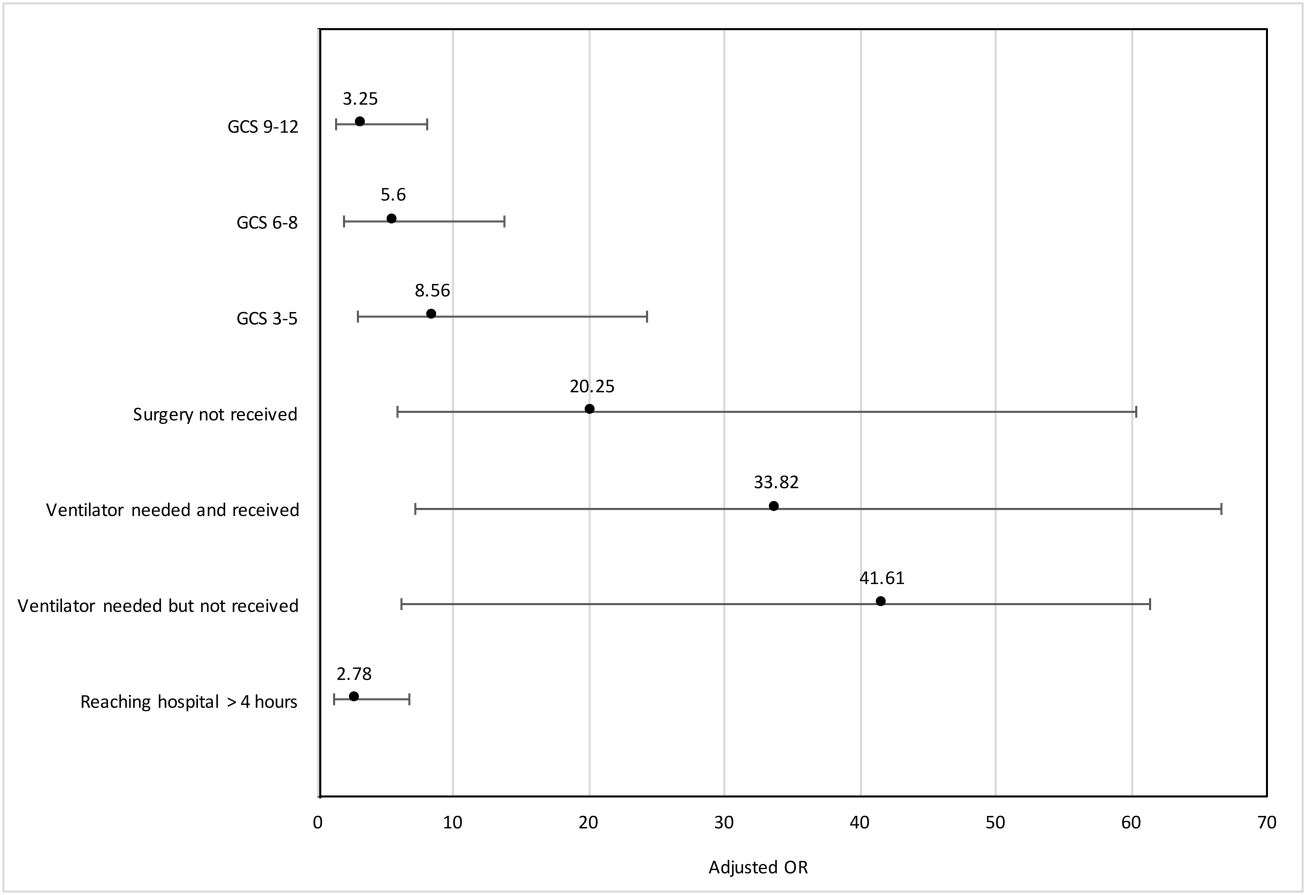
Multivariate model of predictors of traumatic brain injury mortality by adjusted odds ratios (OR). This parsimonious multivariate model was generated through logistic regression using the stepwise backward elimination approach at the p<0.05 level. The model was adjusted for the following confounders: age, polytrauma, and CT diagnosis.

While acute subdural hematoma, more than one intracranial bleed, and more than one minor pathology without intracranial bleed were not significant following multivariate modelling, they were still controlled for in the final model (Table 2 and Fig 3).

Eight of the thirteen clinical assessment variables were associated with mortality on univariate analysis. In decreasing order of unadjusted OR, they are: GCS 3–5, abnormal pupillary exam, cranial nerve deficit, incontinence, limb weakness, GCS 6–8, drops in GCS of 2 or more, and GCS 9–12. Following multivariate analysis, only TBI severity (GCS 3–5 aOR 8.6, 3.0–24.3, GCS 6–8 aOR 5.6, 2.0–13.8, GCS 9–12 aOR 3.3, 1.4–8.0) was retained (Table 3 and Fig 3).

Although two patient risk factors were associated with mortality on univariate analysis, oxygen support and advanced airway on initial assessment, they were not significant in the multivariate analysis (Table 4). Among the care continuum variables, only reaching care > 4 hours was retained after multivariate analysis (aOR 2.8, 1.1–6.8) (Table 5 and Fig 3).

All nine of the management variables were associated with mortality on univariate analysis. In decreasing order of unadjusted OR, they are: ventilator support, ICU need, failure to receive surgery, ICU received, continuous BP and pulse monitoring, HDU care, ventilator disruption, any morbidity, and physiotherapy received. In multivariate modelling, two management variables remained significant in predicting mortality: surgery intended but not received (aOR 20.3, 5.8–60.4) and ventilator support needed outside of surgery (received aOR 33.8, 7.2–66.6 and not received aOR 41.6, 6.2–61.3) (Table 6 and Fig 3).

## Discussion

### Summary

To our knowledge, this is the first prospective TBI registry established in Uganda spanning eight variable categories to understand the biggest drivers for poor outcomes in TBI patients and to highlight areas for quality improvement. Within the 6-month study period, 563 patients presented to MNRH with acute TBI predominantly from RTC (62%). 102 patients (18%) received surgery, and 8 died postoperatively (8%). 29 (5%) patients failed to receive surgery due to infrastructural limitations, of which 18 died (62%). 251 patients (45%) received non-operative management, with a mortality rate of 7.6%. 28 variables were significantly associated with mortality on univariate analysis, and 9 variables remained significant on multivariate analysis: moderate to severe TBI (admission GCS 3–5, 6–8, and 9–12), more than one intracranial bleed, surgery intended but not received, HDU care, ventilator support needed and received or not received, and reaching care in greater than 4 hours.

### Benchmarking against high income countries

In-hospital mortality for TBI patients was 9.6%, which is comparable to most contemporary studies in high income countries (HIC).[14,15] ICU and ventilator status were associated with dramatically increased mortality: 60% and 67%, respectively. Moreover, mortality was 40% for patients in need of ventilator support outside of surgery who did not receive it. In comparison, studies in the United States and Italy have reported 26.5% and 17.2% mortality for TBI patients admitted to the ICU, respectively.[16,17] A separate United States study in patients with severe isolated TBI identified mechanical ventilation as a modifiable risk factor for in-hospital mortality.[13] Comparable Kenyan, Nigerian, and Tanzanian studies have found 54%, 52.2%, and 47% mortality, respectively[18–20], with the Nigerian study additionally reporting poorer outcomes for patients receiving mechanical ventilation. When this study was conducted, MNRH had three to four functional ventilators, all located in the six-bed ICU, thereby providing an opportunity for targeted quality initiatives. Additional research is needed to understand the baseline factors of patients receiving such services and the most effective utilization to improve patient outcomes.

We found that lack of access to timely surgical intervention was also associated with increased mortality. Surgical patients had the lowest mortality of all management pathways. However, when significant provider delays and infrastructural bottlenecks prevent patients from receiving timely surgery, mortality soars from 7.8% to 62%. Based on clinical assessment and diagnostics, these patients were admitted to the neurosurgical ward and planned for surgery. However, due to diagnostic delays, cost, limited theatre space, or other factors, they died preoperatively.

The impact of timely surgery is especially apparent in mild TBI patients whose mortality rises from 3.1% when operated to 37.5% when surgery was intended but not delivered. Of the 11 mild TBI patient deaths, four had skull fractures, three had epidural hematomas, one had a subdural hematoma, one had a normal CT, and two awaited CT results. Three experienced a drop in GCS of two or more from admission to next recording and one only had an admission GCS recorded. Half were managed non-operatively, two were postoperative, and three awaited surgery. The causes of death were sepsis (n = 2), meningitis (n = 1), aspiration (n = 1), and unknown (n = 7). Given the limited clinical documentation for this group, poor monitoring and management of neurological status deterioration may contribute to mortality. Standardized clinical documentation coupled with further research is necessary to identify gaps in the management of mild TBI patients and the role of timely surgery.

Prior HIC studies have shown a drop in mortality by 50% in patients who underwent craniotomy or hematoma drainage within four hours of arrival to the emergency department[21], highlighting the importance of timely clinical assessment and neurosurgical intervention in TBI patients. At MNRH, the importance of timely CT imaging is further emphasized in severe TBI patients of which nearly one in three are not admitted to neurosurgery due to the lack of CT scan. Of those not admitted to neurosurgery, nearly half died in-hospital, suggesting an even higher actual mortality rate, as post-hospital outcomes are not recorded. Interestingly, obtaining CT scan within 4 hours of arrival had a 7.2 OR of death compared to delayed CT findings, which suggests that sicker patients may be triaged first, but without improved outcomes. Therefore, further research is needed to understand the magnitude, causes, and impact of delays in MNRH.

Similarly, pre-hospital delays can negatively impact patient outcomes. Delays in seeking care longer than 24 hours and in reaching care longer than four hours were strongly associated with high mortality. To address delays in seeking care, future interventions may target patient education with respect to the time sensitive nature of TBI. Delays in reaching care point to infrastructural factors such as limited access to emergency vehicles, navigating unpaved roads and public transportation, and highly congested traffic. The strong association between transport delays and poor outcomes emphasizes the need for future research and interventions aimed at identifying and improving infrastructural bottlenecks.

### Predictors of mortality

Factors predictive of TBI mortality in Uganda can be divided into delays in reaching care, injury severity (GCS<13), and hospital interventions (surgery and step-up care). As previously discussed, timely access to neurosurgical intervention is essential for TBI patients, therefore delays in reaching care negatively impact patient outcomes. Patient baseline and injury risk factors are consistent with previous studies which report worse outcomes for patients with more severe TBI.[14,22] Similarly, over half of patients with more than one intracranial bleed had moderate to severe TBI.

With respect to hospital interventions, the classification of a patient for whom surgery was intended but not received proved to be highly predictive of mortality. These patients experienced a **more than tenfold mortality than all other patients**. One reason they were not operated is their choice to self-discharge against medical advice. Therefore, the high mortality rate reported may be an underestimation of the true mortality rate, since patient outcomes post-discharge are unknown. For patients dying in the hospital prior to their surgery, reasons for surgical delays are multifaceted, including limitations in theatre space, delayed procurement of supplies, and diagnostic logistics. The dramatic increase in mortality for patients not receiving necessary surgical care stresses the importance of increasing access to neurosurgery in future interventions to address morbidity and mortality.

With respect to diagnostic capacity, patients at MNRH are faced with challenges in access and cost similar to patients in other LMIC settings.[23] To obtain a head CT scan—necessary for surgical decision making—caregivers typically arrange their own transportation to nearby private Nakasero Hospital, the only facility within close proximity to MNRH with a functional CT scanner, and pay for the scan and films out of pocket. The financial burden of a CT scan, which can range from $70 to $132[24], can be almost equal to the average monthly income in Uganda.[25] Further investigation into the causes of surgical delays is necessary to guide future interventions.

Similarly, receiving step-up care through HDU admission and mechanical ventilation was predictive of mortality. Although ICU admission was not retained as a predictive factor in the multivariate analysis, the co-linearity between ICU care and mechanical ventilation, coupled with the strong association between ICU admission and high mortality, emphasize the opportunity for targeted interventions in step-up care to impact patient outcomes.

### Limitations

In an effort to provide a comprehensive perspective on TBI patient management at MNRH, 60 variables throughout the care continuum are tracked per patient. As a result, this study was limited by the ability to collect complete data for all patients throughout their hospital stay. Moreover, the quality of data collection was limited by availability of clinical documentation. For example, only 17% of patients had admission vitals recorded in the patient charts, with the proportion of the other 83% that were not assessed or assessed but not recorded unknown. To mitigate the burden on clinical staff, data collection forms were printed and placed in the patient charts. However, there were still several variables predominantly from the admission vitals category that had more than 20% missing data. Additionally, because the RAs are not clinically trained, they are unable to collect missing data points such as admission vitals not documented in the charts and may misclassify certain risk factors from clinical notes.

During this study prior to surgery, patients will need to have CT scans done outside of MNRH at the private facility Nakesero hospital, which had the only functional CT scanner. This presents a significant logistical challenge for timely clinical intervention and explains the variation in processes of care. Specifically, this initial registry development phase identified that nearly 20% (N = 111) of patients did not receive CT scans due to financial and logistical reasons. Consequently, the 8.1% mortality (N = 9) for this group is likely an underestimate. Understanding the barriers to CT scan access in future studies will be crucial for quality improvement initiatives and have potential health policy implications.

Due to the limited sample size in this first 6-month report (N = 563; Deaths = 54), logistic regression to identify significant risk factors for poor outcomes was performed in one model. The model controlled for the following confounders: age, polytrauma, and CT diagnosis. GCS was used as a measure of risk stratification and its adjusted ORs were reported. However, specific risk adjustments with Injury Severity Score (ISS) and Sequential Organ Failure Assessment (SOFA) score that highlight the impact of polytrauma and extensive multisystem comorbidity were not possible since they are not routinely collected.

### Future studies

This study provides a framework for future TBI studies and registry development for other patient populations in LMIC. Review of our prospective neurosurgical registry estimates the burden of TBI to account for 80% of patients managed by the neurosurgery department at MNRH. As such, future studies may expand to include all neurosurgical patients at MNRH. The patient outcomes presented in this study also call for further research into the underlying infrastructural and systems-based factors affecting mortality. In these efforts, to mitigate the burden on clinical staff, data collection The drastically high mortality in patients for whom surgery was intended but not received warrants further exploration into the causes of surgical delays. By identifying bottlenecks in the care continuum, future interventions may address infrastructural or provider delays affecting patient outcomes. Moreover, the prospective nature of the registry allows for up-to-date statistics on the management of TBI patients at MNRH to the clinical team. In a setting where patient records are in paper format, regular and timely reports based on an electronic registry may provide high-level insights to the clinical team that would otherwise be unavailable.

Previous studies have reported the use of Kampala Trauma Score (KTS) in risk adjustment[10], but this was not consistently noted in the TBI patient files. In the next phase to strengthen the registry and complement local clinical practice, future studies should aim to integrate additional relevant risk adjustment variables such as ISS, a modified SOFA score, or KTS. Furthermore, these quality improvement initiatives, including this TBI registry, should promote cross talk across departments for improved standardized protocols.

While there is increasing literature describing the disproportional burden of TBI in LMICs, its epidemiological pattern, and increased surgical capacity programs, there remains a need to systematically examine how demographics, diagnostics, patient risk profiles, clinical data, care continuum delays, and management pathways interrelate and affect patient outcomes. Our prospective study examined 563 TBI patients across 8 variable categories presenting to MNRH over a 6-month period to determine significant predictors of poor outcomes and highlight areas for improvement and further research. Although the mortality rate is comparable to other LMICs, it ranged from 3–82% depending on the intervention type, its availability, and disease severity, with tenfold increase in mortality for patients intended for surgery who failed to receive it. As TBI burden remains consistently high with a complex interplay of factors contributing to the heterogeneous mortality rate, further research and collaborative efforts in complementing better resource allocation practices to existing neurosurgical residency education are imperative.

## Supporting information

S1 Data Data used to generate results. (XLS)
